# Effects of Virtual Reality-Based Intervention on Cognition, Motor Function, Mood, and Activities of Daily Living in Patients With Chronic Stroke: A Systematic Review and Meta-Analysis of Randomized Controlled Trials

**DOI:** 10.3389/fnagi.2021.766525

**Published:** 2021-12-13

**Authors:** Yong Gao, Lu Ma, Changsheng Lin, Shizhe Zhu, Lingling Yao, Hong Fan, Jianqiu Gong, Xiaobo Yan, Tong Wang

**Affiliations:** ^1^Department of Rehabilitation, Shaoxing People’s Hospital (Shaoxing Hospital, Zhejiang University School of Medicine), Shaoxing, China; ^2^Library, Zhejiang Industry Polytechnic College, Shaoxing, China; ^3^School of Rehabilitation Medicine, Nanjing Medical University, Nanjing, China; ^4^Department of Rehabilitation, The First Affiliated Hospital of Nanjing Medical University, Nanjing, China

**Keywords:** cognition, motor, virtual reality, chronic stroke, meta-analysis

## Abstract

**Background:** The efficacy of virtual reality (VR)-based intervention for improving cognition in patients with the chronic stage of stroke is controversial. The aims of this meta-analysis were to evaluate the effect of VR-based training combined with traditional rehabilitation on cognition, motor function, mood, and activities of daily living (ADL) after chronic stroke.

**Methods:** The search was performed in the Cochrane Library (CENTRAL), EBSCO, EMBASE, Medline (OVID), Web of Science databases, PubMed, CINAHL Ovid, and Scopus from inception to May 31, 2021. All included studies were randomized controlled trials (RCTs) examining VR-based intervention combined with traditional rehabilitation for chronic stroke. The main outcomes of this study were cognition, including overall cognition (combined with all cognitive measurement results), global cognition (measured by the Montreal Cognitive Assessment, MoCA, and/or Mini-Mental State Examination, MMSE), and attention/execution. The additional outcomes were motor function, mood, and ADL. Subgroup analyses were conducted to verify the potential factors for heterogeneity.

**Results:** Six RCTs including 209 participants were included for systematic review, and five studies of 177 participants were included in meta-analyses. Main outcome analyses showed large and significant effect size (ES) of VR-based training on overall cognition (*g* = 0.642; 95% CI = 0.134–1.149; and *P* = 0.013) and attention/execution (*g* = 0.695; 95% CI = 0.052–1.339; and *P* = 0.034). Non-significant result was found for VR-based intervention on global cognition (*g* = 0.553; 95% CI = −0.273–1.379; and *P* = 0.189). Additional outcome analyses showed no superiority of VR-based intervention over traditional rehabilitation on motor function and ADL. The ES of VR-based intervention on mood (*g* = 1.421; 95% CI = 0.448–2.393; and *P* = 0.004) was large and significant. In the subgroup analysis, large effects for higher daily intensity, higher weekly frequency, or greater dose of VR intervention were found.

**Conclusion:** Our findings indicate that VR-based intervention combined with traditional rehabilitation showed better outcomes for overall cognition, attention/execution, and depressive mood in individuals with chronic stroke. However, VR-based training combined with traditional rehabilitation showed a non-significant effect for global cognition, motor function, and ADL in individuals with chronic stroke.

## Introduction

Stroke is one of the global leading causes of death and may cause long-term disability for many stroke survivors (Mendis, 2013; Andrew et al., 2014). Up to three-quarters of patients with poststroke experienced ongoing cognitive impairment (Pasi et al., 2012; Jokinen et al., 2015; Renjen et al., 2015). Cognitive impairment and functional disability are often associated with the following stroke. Furthermore, the depressive mode worsens the difficulties for patients with stroke to maintain their social and personal relationships. Clinical depression is characterized by behavioral, cognitive, and emotional features (Merriman et al., 2019). Cognitive performance is always associated with symptoms of depression (Nakling et al., 2017), and early cognitive deficits in patients after stroke may predict long-term depressive symptoms (Nys et al., 2006). Furthermore, poststroke cognitive impairment is associated with early and enduring activity limitations and participation restrictions (Stolwyk et al., 2021). These disorders might lead to a poor quality of life (QoL) for individuals with stroke and their families.

In recent years, interventions for poststroke motor and cognitive impairment, depression, and reduced functional independence have become the focus of international stroke rehabilitation research, and novel clinical rehabilitation therapies [e.g., virtual reality (VR), repetitive transcranial magnetic stimulation (rTMS), and robotic assistive therapies] have shown great potential in future practice (Langhorne et al., 2011; Winstein et al., 2016a; Gittler and Davis, 2018). VR-based training is defined by using computer hardware and software-generated user-computer interface for users to interact with virtual environments that relate to the real world to facilitate task-oriented training and provide multimodal feedback to augment functional recovery (Laver et al., 2017; Hao et al., 2021). Basic neuroscience behind VR-based treatment was the finding of mirror neurons (MNs) in the primary motor cortex (M1), dorsal premotor cortex, and supplementary motor area (SMA) from animal studies (Gentilucci et al., 1988; Rizzolatti et al., 1996; Rizzolatti and Sinigaglia, 2016; Mekbib et al., 2020). The evidence from human neuroimaging suggested that the neural mechanisms of VR on neural plasticity and motor reorganization in humans might be to stimulate the internal sensorimotor system through activating MNs in the cortical and subcortical motor control-related areas, particularly M1, SMA, and cerebellum (August et al., 2006(Prochnow et al., 2013; Mekbib et al., 2020, 2021; Hao et al., 2021).

Recently, many clinical studies favored VR-based intervention for motor function, balance, gait, and activities of daily living (ADL) in patients with stroke. Although multiple systematic reviews and meta-analyses have indicated that VR-based training was useful for upper limb motor function, lower limb motor function, balance, gait, and activities of daily living (ADL) in stroke (Henderson et al., 2007; Laver et al., 2011; Saposnik et al., 2011; Lohse et al., 2014; Laver et al., 2015; de Rooij et al., 2016; Li et al., 2016; Silver, 2016; Yates et al., 2016; Laver et al., 2017; Aminov et al., 2018; Al-Whaibi et al., 2021; Fang et al., 2021; Peng et al., 2021; Zhang et al., 2021), two recent articles published in *The Lancet Neurology* by Saposnik et al. (2016) and Silver (2016) argued that the methodological issues that existed in some of the studies (Broeren et al., 2008; Kwon et al., 2012) were the comparison of VR combined with conventional rehabilitation vs. conventional rehabilitation alone without active control. Such study design (Saposnik et al., 2011; Lohse et al., 2014; Laver et al., 2015) might create an imbalance in the total rehabilitation time, and the effect might be induced by any active intervention and might not be explained by VR (Saposnik et al., 2016; Silver, 2016).

Conventional paper-and-pencil exercises and computer-assisted cognitive training designed to improve specific domains of cognitive deficits are widely used for patients with stroke with cognitive impairment. However, traditional cognitive training is limited by its insufficient personalization and adaptation and suboptimal intensity (Faria et al., 2016; Maier et al., 2020). Preliminary results (Kim et al., 2011; Choi et al., 2014; Faria et al., 2016, 2020; De Luca et al., 2018; Kannan et al., 2019; Maggio et al., 2019; Oh et al., 2019; Maier et al., 2020; Manuli et al., 2020) suggested that VR-based training combined with traditional rehabilitation might be more effective for enhancing cognition, depressive mood, and QoL in stroke than traditional cognitive rehabilitation. However, there is no clear evidence concerning the effectiveness of VR for cognition, depression, and QoL in patients with stroke (Laver et al., 2011, 2015; Silver, 2016). Recently, several systematic reviews (Aminov et al., 2018; Wiley et al., 2020; Zhang et al., 2021) have evaluated the effectiveness of VR for cognitive impairment in patients with stroke. Aminov et al. (2018) included 4 studies that assess VR-based rehabilitation on cognitive outcomes and found that VR could induce significant gains on improvements in cognitive function. Zhang et al. (2021) combined 7 RCTs to evaluate the effectiveness of VR interventions for cognitive outcomes compared with control groups, but no significant difference was found. However, in the two meta-analyses (Aminov et al., 2018; Zhang et al., 2021), only global cognition examined by MMSE or MoCA test for screening cognitive impairment was included, and specific domains of cognition were not investigated. Wiley et al. (2020) performed a systematic review that included five manuscripts to evaluate VR-based intervention combined with rehabilitation exercise on global cognition and specific domains of cognition and concluded that VR therapy was not better than traditional rehabilitation interventions for enhancing cognitive function in stroke survivors. However, due to the limited number of original articles, small sample size, different types of VR devices, different VR intervention durations, and different stages after stroke onset, the results remain controversial.

To date, however, few systematic reviews and meta-analyses have investigated VR-based training for cognitive function in contrast to cognitive exercise or motor exercise on the chronic stage of stroke. Therefore, this study aimed to explore the effect of VR-based training on cognition, motor function, mood, and ADL among individuals at the chronic phase of stroke.

## Materials and Methods

### Search Strategy and Eligibility Criteria

The current meta-analysis was conducted and reported in accordance with the Preferred Reporting Items for Systematic Reviews and Meta-Analysis (PRISMA) guidelines.

Systematic search was performed using electronic databases such as Cochrane Library (CENTRAL), EBSCO, EMBASE, Medline (OVID), PubMed, CINAHL Ovid, Scopus, and Web of Science databases from inception to May 31, 2021. Boolean search terms included the following: “cerebrovascular accident (CVA),” “stroke,” “VR” with different combinations, and associated Medical Subject Headings. The specific search syntax (e.g., web of science) is available in Supplementary Appendix 1.

We also hand-searched the reference lists from relevant reviews and articles to identify any potentially relevant studies.

In this review, randomized controlled trials (RCTs) that examined the effects of VR on cognition, motor function, mood, or ADL in patients with chronic stroke were included. Eligibility criteria were formulated based on the PICOS framework (Hutton et al., 2016): (1) Participants: subjects aged above 18 years and evaluated for a period of over 6 months after diagnosis of stroke. (2) Intervention: The VR interventions should be based on standardized computerized task-oriented therapies or interactive video games. (3) Control: the comparison group should be motor and/or cognitive therapies that did not use VR-based devices. If one trial included three or more groups, then the group that received VR intervention plus traditional rehabilitation was chosen as the experimental group, and the group that only received traditional rehabilitation was chosen as the control group for this study. (4) Outcome measures: the main outcomes are cognitive function, including overall cognition (combined with all cognitive measurement results), global cognition (measured by the Montreal Cognitive Assessment, MoCA, or Mini-Mental State Examination, MMSE), and attention/execution. The additional outcomes are motor function, mood, and activities of daily living. All included outcome measures should be evaluated both at the onset of the intervention and at the end of the intervention. Further data analyses of outcomes at follow-up were not included. (5) Study: eligible studies were RCTs published in peer-reviewed journals that investigated the efficacy or effectiveness of VR-based rehabilitation on one or more domains of cognition with or without motor function, mood, and ADL in the chronic phase of stroke. The following types of studies were excluded: individuals with visual impairment, graduation theses, books, conference abstracts, case reports, prospective or retrospective cohort studies, full texts cannot be reached, data cannot be extracted, and not written in English.

### Selection of Studies

Two independent reviewers (YG and LM) screened the retrieved titles, abstracts, and full texts for eligibility according to their relevance. Full-text evaluations and data extraction were performed where abstracts did not provide sufficient information. The respective authors were contacted by mail if the information available was incomplete or any obscurities were present. Disagreements regarding study eligibility were resolved by consensus after a discussion.

### Data Extraction and Quality Assessment

Data including general characteristics (e.g., first author, country, and study design), patient characteristics (e.g., sample size, stroke type, affected extremity, mean time poststroke, and mean age), intervention characteristics (e.g., intervention intensity, duration, type of VR device, and interactive media) in each trial were extracted. The intervention design and the difference in the two groups and main outcome measures were also summarized and compared. Active interventions were defined for the control group receiving the same total training time as the experimental group, while passive interventions were defined as a blank control group.

The Physiotherapy Evidence Database (PEDro) Scale (Maher et al., 2003) was used to assess the quality of each included trial. We included studies that scored six or higher with the PEDro Scale for their high quality. The Cochrane Collaboration’s tool (Higgins et al., 2011) was used to evaluate the methodological quality of the included studies. The scoring process was conducted by two authors (YG and LLY), and any disagreements were resolved by consensus or a third investigator.

### Statistical Analysis

The Comprehensive-Meta-Analysis software package (version 2.0, Biostat, Inc., Englewood, NJ, United States) was used to perform the meta-analysis. Means and SD between groups from baseline to immediately after intervention were reported as provided by the authors, estimated from a graph or from the medians and IQR. Studies were excluded from meta-analysis if data estimated from a graph or from the medians and IQR were significantly skewed away from normality. The Hedges’ *g* was used to quantify the efficacy of VR-based training. The effect size (ES) was categorized as follows: small (<0.3), medium (≥0.3 and <0.6), and large (≥0.6) (Hill et al., 2017). ES outcomes were positive if postintervention performance was better than baseline performance. We assessed heterogeneity using the *I*^2^ statistic, where an *I*^2^ value greater than 50% indicated significant heterogeneity (Higgins et al., 2003). The pooled treatment effect of the individual studies that were combined was evaluated by a random-effects model to reduce the effects of heterogeneity between studies. Subgroup analyses were conducted, including the dose of intervention, intervention daily intensity, intervention frequency, intervention sessions, cognitive task, and additional therapy.

## Results

### Literature Review

**FIGURE 1:**
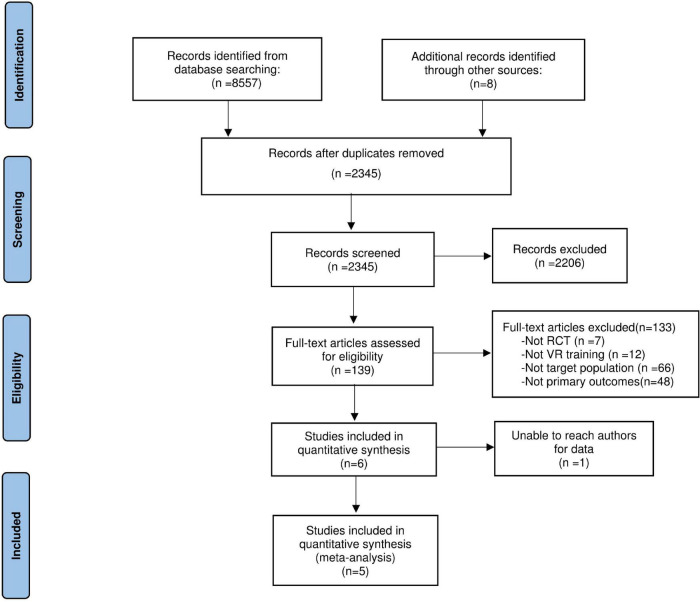
Preferred reporting items for systematic reviews and meta-analysis (PRISMA) flowchart for study selection.

The flow diagram of identifying eligible trials is outlined in Figure 1. Among 8,557 articles from eight databases and eight additional RCTs which were searched through other sources, 139 potentially relevant studies were retrieved. Over two researchers independently examined the full texts of the 139 potential articles, 6 trials were included in the literature review. One study (Faria et al., 2020) was excluded for quantitative synthesis, for the outcome scores for some data were significantly skewed away from normality. Another study (Manuli et al., 2020) included three groups, then the group that received VR intervention plus traditional rehabilitation was chosen as the experimental group, and the group that only received traditional rehabilitation was chosen as the control group for this study. The characteristics of each RCT included are presented in Table 1. Among the 6 included articles, 4 articles (66.7%) were from Europe, 1 article (16.6%) was from the United States, and 1 article (16.6%) was from Korea. The median number of participants for the included articles was 32 (range: 24–60). Four trials (66.7%) reported stroke type (both ischemic stroke and hemiplegic stroke), while two trials (33.3%) did not report specific stroke type. Four trials (66.7%) reported affected extremity, while two trials (33.3%) did not report affected extremity. All studies reported the mean time poststroke. The VR devices reported in all included studies were semi-immersive VR. The comparison of interventions in each study is shown in Table 2. The outcome measures for cognition, motor function, mood, and ADL are listed in Table 3.

**TABLE 1:** Study characteristics of the included trials.

Author(s), year	Country of origin	Study design	Sample								VR intervention design					PEDro score
			N (EG/CG)	Stroke type		Affected extremity		Mean (SD), time post-stroke (mo)	Age (year), mean (SD)	Sex (male) (%)	Session length/ Per week/N	Cost of time	Immersive type	VR content	Interactive medium	
				Hem	Isch	Left	Right									
Manuli et al. (2020)	Italy	RCT	60 (30/30)	NR	NR	EG:5 CG:5	EG:25 CG:25	EG:135.0 (45.0) CG:126.0 (30.0)	EG:48.0 (12.1) CG:40.1 (10.7)	EG:19 (63.3) CG:6 (20.0)	60 min/ 5/40	40 h	Semi	Task	Motion tracking	9
Maier et al. (2020)	Spain	RCT	38 (19/19)	EG:7 CG:5	EG:12 CG:14	NR	NR	EG:28.37 (26.84) CG:420.86 (45.87)	EG:63.63 (6.73) CG:67.21 (6.45)	EG:11 (57.89) CG:12 (63.16)	30 min/ 5/30	15 h	Semi	Task	Kinect/motion track sensor	7
Faria et al. (2020)	Portugal	RCT	32 (14/18)	EG:2 CG:3	EG:12 CG:15	EG:3 CG:6	EG:11 CG:12	EG:45.93 (43.56) CG:21.33 (12.88)	EG:59.14 (11.81) CG:65.00 (6.20)	EG:5 (35.71) CG:11 (61.11)	30 min/ 3/12	6 h	Semi	Task	Reh@City/Motion tracking	7
Oh et al. (2019)	Korea	RCT	31 (17/14)	NR	NR	NR	NR	NR	EG:57.4 (12.2) CG:52.6 (10.7)	EG:12 (38.7) CG:9 (29.0)	30 min/ 3/18	9 h	Semi	Task	Joystim	6
Kannan et al. (2019)	United States	RCT	24 (13/11)	EG:5 CG:5	EG:8 CG:6	EG:6 CG:6	EG:7 CG:5	EG: 106.8 (64.73) CG: 109.1 (76.32)	EG: 57.5 (8.04) CG: 61.0 (4.60)	EG:7 (53.8) CG:6 (54.5)	90 min/ 2–5/20	30 h	Semi	Task	Wii Fit	7
Faria et al. (2018)	Portugal	RCT	24 (12/12)	EG:11 CG:9	EG:1 CG:1	EG:8 CG:7	EG:4 CG:5	EG:24.9 (20.3) CG:41.1 (41.0)	EG:57.1 (11.0) CG: 68.9 (9.8)	EG:8 (62.7) CG:7 (58.3)	45 min/ 3/12	9 h	Semi	Task	Motion tracking	7

*EG, experimental group; CG, control group; VR, virtual reality; N, number; NR, not reported; RCT, randomized controlled trial; Isch, ischemic; Hem, hemorrhagic; wk, week; mo, months.*

**TABLE 2:** Characteristics of interventions in included studies.

Study	Experimental group intervention	Control group intervention	Follow-up	Effectiveness of control group
Manuli et al. (2020)	Rehabilitation training with the Lokomat-Pro with a virtual reality (VR)-screen 60 min × 5 session a week for 8 wk plus physiotherapy 60 min × 5 session a week for 8 wk	Rehabilitation training with the Lokomat Nanos 60 min × 5 session a week for 8 wk plus physiotherapy 60 min × 5 session a week for 8 wk OR conventional physiotherapy and cognitive treatment 180 min × 5 session a week for 8 wk	0 and 8 wk	Active
Maier et al. (2020)	adaptive conjunctive cognitive training (ACCT) using a VR-based rehabilitation tool, Rehabilitation Gaming System (RGS) 30 min × 5 session a week for 6 wk	A folder with 30 individual cognitive tasks selected by the neuropsychologist to overlap with the cognitive abilities essential in the experimental tasks 30 min × 5 session a week for 6 wk	0, 6, and 18 wk	Active
Faria et al. (2020)	adaptive cognitive training through everyday tasks VR simulations the Reh@City v2.0 30 min × 3 session a week for 4 wk	adaptive paper-and-pencil training generated automatically through a Task Generator 30 min × 3 session a week for 4 wk	0, 4, and 8wk	Active
Kannan et al. (2019)	Wii-fit games in conjunction with cognitive tasks 90 min × 2–5 session a week for 6 wk	customized, progressive balance training 90 min × 2–5 session a week for 6 wk	0, 7, and 11 wk	Active
Oh et al. (2019)	Joystim for the VR combined with real instrument training 30 min × 3 session a week for 6 wk	conventional occupational therapy 30 min × 3 session a week for 6 wk	0, 6, and 10 wk	Active
Faria et al. (2018)	Training with the Reh@Task virtual cognitive-motor task, which combines adapted arm reaching, and attention and memory training 45 min × 3 session a week for 4 wk plus conventional occupational therapy 45–60 min × 2–3 session a week for 4 wk	time-matched conventional occupational	0, 1, and 2 mo	Active

*wk, weeks; mo, month.*

**TABLE 3:** Outcome measures assessing VR in patients with chronic stroke.

Study	Attention/ Execution	Global cognition	Motor	Mood	ADL
Manuli et al. (2020)	WEIGL	MoCA		BDI-II	SF-12 FIM
	FAB				
	VS				
	TMT				
Maier et al. (2020)	Corsi-F	MoCA	FMA-UE	HAM-D	BI
	TMT-A	MMSE			
	WAIS-F				
	TMT-B				
	WAIS-C				
	FAB				
Faria et al. (2020)	TMT-A	MoCA			
	TMT-B	PRECiS			
	WMSIII-DS				
	WAISIII-SS				
	WAISIII-DSC				
Kannan et al. (2019)	Cognitive training-performance scores				
	LNS- accuracy				
	Word List Generation-accuracy				
Oh et al. (2019)		K-MMSE	FMA-UE		
		K-MoCA	BBT		
			hand grip 9-HPT		
Faria et al. (2018)	Cancelation Tests-SLC	MoCA	FMA-UE		BI
	Cancelation Tests-DC		CAHAI		
	Cancelation Tests-BT				

*WEIGL, Weigl test; FAB, frontal assessment battery; VS, visual search; TMT, trail making test; MoCA, Montreal cognitive assessment; BDI II, beck depression inventory-II; SF-12, short form-12; FIM, functional independence measure; Corsi F, Corsi block tapping test forward; TMT-A, trail making test form A; WAIS F, Wechsler Adult Intelligence Scale-Digit Span Forward; TMT-B, trail making test form B; WAIS C, WAIS digit symbol coding; Corsi B, Corsi block tapping test backward; RAVLT I, Rey Auditory Verbal Learning Test Immediate; RAVLT D, Rey Auditory Verbal Learning Test Delayed Recall; MMSE, Mini-Mental State Examination; HAM-D, Hamilton Depression Rating Scale; WAISIII, Wechsler Adult Intelligence Scale III; DS, digit span; SS, symbol search; DSC, digit symbol coding; WMS-III, Wechsler Memory Scale-III; VPA, verbal paired associates; PRECiS, patient-reported evaluation of cognitive state; Corsi F, Corsi block tapping test forward; LNS, letter number sequencing; K-MMSE, Korean-Mini-Mental State Examination; Korean-Montreal Cognitive Assessment (K-MoCA); FMA-UE, Fugl-Meyer Assessment Test-Upper Extremity; BBT, box and block test; 9-HPT, 9-Hole Peg Test; SLC, single letter cancelation; DC, digit cancelation; BT, Bells Test; CAHAI, Chedoke Arm and Hand Activity Inventory; BI, barthel index.*

### Study Quality and Risk of Bias

**FIGURE 2:**
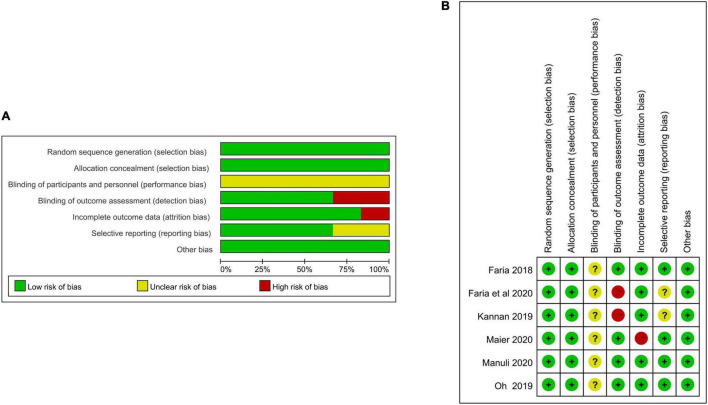
**(A)** Assessment of risk of bias with selected studies. **(B)** Risk of bias graph and summary.

The study quality of the included RCTs is shown in Table 1. Six included studies for systematic review were of high quality for PEDro score, and the mean score for the PEDro scale was 7.17. Table 4 and Figure 2 show the risk of bias of the included RCTs. All studies had a low risk of bias on sequence generation, allocation concealment, outcome assessor blinding, selective outcome reporting, and other sources of bias (Faria et al., 2018, 2020; Kannan et al., 2019; Oh et al., 2019; Maier et al., 2020; Manuli et al., 2020). All studies had unclear bias on therapist and participant blinding (Faria et al., 2018, 2020; Kannan et al., 2019; Oh et al., 2019; Maier et al., 2020; Manuli et al., 2020). One study had a high risk of bias on incomplete outcome data (Maier et al., 2020).

**TABLE 4:** Risk of bias assessed for all included studies.

References	Sequence generation	Allocation concealment	Blinding		Incomplete outcome data	Selective Outcome reporting	Other sources of bias
			Therapist and participants	Outcome assessors			
Manuli et al. (2020)	Low risk	Low risk	Unclear	Low risk	Low risk	Low risk	Low risk
Maier et al. (2020)	Low risk	Low risk	Unclear	Low risk	High risk	Low risk	Low risk
Faria et al. (2020)	Low risk	Low risk	Unclear	Low risk	Low risk	Low risk	Low risk
Oh et al. (2019)	Low risk	Low risk	Unclear	Low risk	Low risk	Low risk	Low risk
Kannan et al. (2019)	Low risk	Low risk	Unclear	Low risk	Low risk	Low risk	Low risk
Faria et al. (2018)	Low risk	Low risk	Unclear	Low risk	Low risk	Low risk	Low risk

### Main Outcome and Additional Outcome Analyses

**FIGURE 3:**
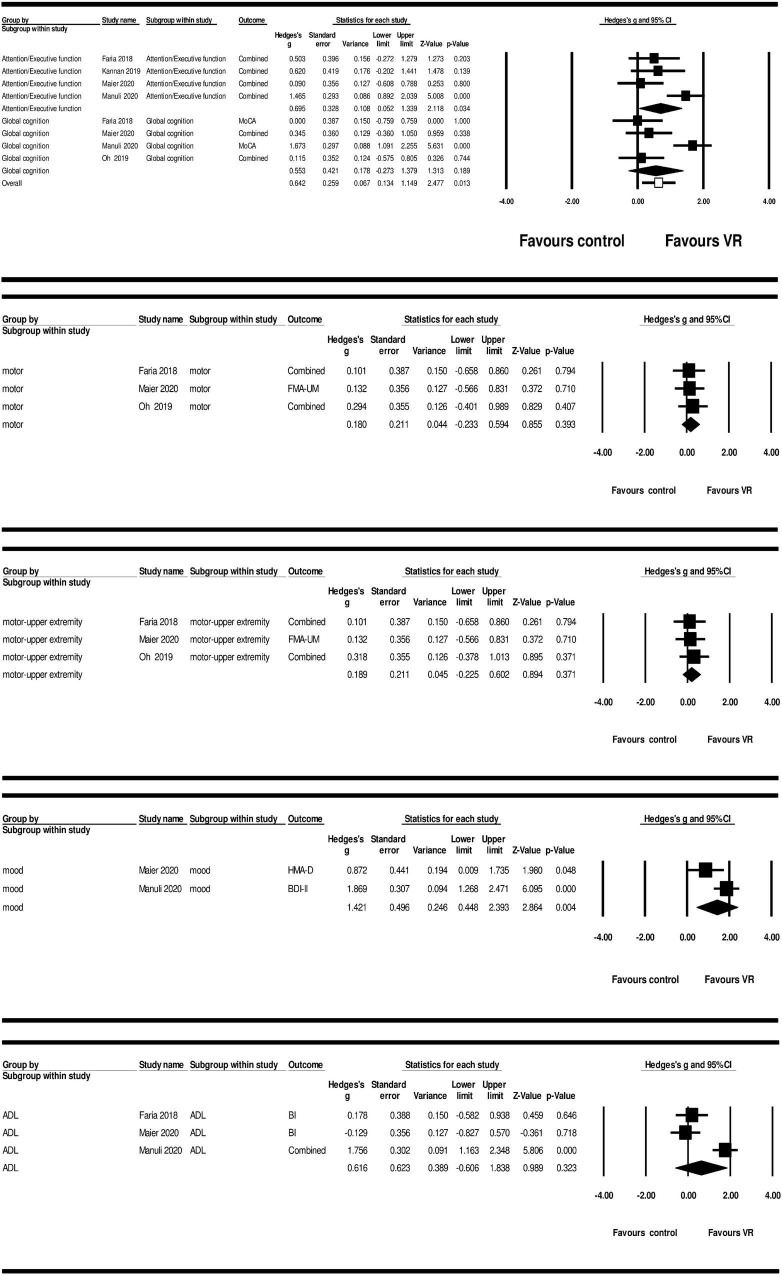
Forest plot showing the main effect-sizes of VR intervention on cognition, motor, motor-upper extremity, mood, and ADL vs. control group in patients with chronic stroke.

Main outcome analyses using a random effects model revealed large and significant ES of VR-based training on overall cognition (*g* = 0.642; 95% CI = 0.134–1.149; *P* = 0.013; and *I*^2^ = 0%) and attention/execution (*g* = 0.695; 95% CI = 0.052–1.339; *P* = 0.034; and *I*^2^ = 0%). Non-significant result was found for VR-based intervention on global cognition (*g* = 0.553; 95% CI = −0.273–1.379; *P* = 0.189; and *I*^2^ = 0%) (Figure 3 and Table 5).

**TABLE 5:** Effectiveness of main and additional outcome measures.

		K	N	ES (Hedges’s g)	Standard error	95% CI	Q	p(Q)	*I* ^2^
Cognitive functions	Global cognition	4	138	0.553	0.421	**−**0.273–1.379	2.599	0.189	0.000
	Execution/Attention	4	138	0.695	0.328	0.052–1.339	2.501	0.034	0.000
	Overall cognition	4	138	0.642	0.259	0.134–1.149	5.170	0.013	0.000
Motor functions	Motor	3	86	0.180	0.211	**−**0.233–0.594	0.162	0.393	0.000
	Upper extremity	3	86	0.189	0.211	**−**0.255–0.602	0.208	0.371	0.000
Mood	Mood	2	81	1.421	0.496	0.448–2.393	1.000	0.004	0.000
Activities of daily living	ADL	3	115	0.616	0.623	**−**0.606–1.838	1.778	0.323	0.000

*ADL, activities of daily living; K, number of studies; N, number of patients; ES, effect size; CI, confidence interval; Q, within domain heterogeneity; P(Q), P-value for heterogeneity; I^2^, percentage of heterogeneity due to true differences within studies.*

Additional outcome analysis also showed non-significant results of VR-based intervention on motor function (*g* = 0.180; 95% CI = −0.233–0.594; *P* = 0.393; and *I*^2^ = 0%), motor-upper extremity (*g* = 0.189; 95% CI = – 0.225–0.602; *P* = 0.371; and *I*^2^ = 0%), and ADL (*g* = 0.616; 95% CI = −0.606–1.838; *P* = 0.323; and *I*^2^ = 0%). However, the ES of VR-based training on mood (*g* = 1.421; 95% CI = 0.448–2.393; *P* = 0.004; and *I*^2^ = 0%) was significant and large (Figure 3 and Table 5).

### Subgroup Analyses

Results of subgroup analysis based on study characteristics are reported in Table 6. For the dose of VR-based intervention, dose ≥ 20 h showed larger and significant ES (*g* = 1.147; 95% CI = 0.206–2.089; and *P* = 0.017) than dose lower than 20 h (*g* = 0.263; 95% CI = −0.156–0.681; and *P* = 0.219). Regarding VR-based intervention frequency, the ES of more than four times per week (*g* = 1.063; 95% CI = 0.611–1.515; and *P* < 0.001) was larger and significant than less than four times per week (*g* = 0.111; 95% CI = −0.312–0.534; and *P* = 0.607). The average ES for VR intervention daily intensity more than 60 min was larger and significant (*g* = 1.147; 95% CI = 0.206–2.089; and *P* = 0.017) than studies with daily intensity less than 60 min (*g* = 0.263; 95% CI = −0.156–0.681; and *P* = 0.219). In terms of the intervention sessions, cognitive task, and additional therapy, non-significant ES was observed for subgroups between VR and control groups with the random effects model.

**TABLE 6:** Effectiveness of subgroup analysis according to study characteristics.

	Categories	K	ES (Hedges’s g)	+95% CI		*p*	SE
Dose of intervention	≥20h	2	1.147	0.206	2.089	0.017	0.480
	<20h	3	0.263	−0.156	0.681	0.219	0.213
Intervention frequency	≥4/wk	2	1.063	0.611	1.515	<0.001	0.231
	<4/wk	3	0.362	−0.074	0.798	0.103	0.222
Intervention daily intensity	≥60 min	2	1.147	0.206	2.089	0.017	0.480
	<60 min	3	0.263	−0.156	0.681	0.219	0.213
Intervention sessions	≥30	2	0.959	−0.346	2.264	0.150	0.666
	<30	3	0.373	−0.077	0.823	0.104	0.230
Cognitive task	Yes	3	0.364	−0.078	0.806	0.107	0.226
	No	2	0.933	−0.370	2.237	0.160	0.665
Additional therapy	Yes	2	0.952	−0.335	2.239	0.147	0.657
	No	3	0.356	−0.070	0.783	0.218	0.102

*K, number of studies; ES, effect size; h, hour; wk, week; min, minute; CI, confidence interval; SE, standard error.*

## Discussion

In the present meta-analysis from five high-quality RCTs, the baseline global cognitive scores all reported mild cognitive impairment, and we showed the superiority of using VR-based intervention combined with rehabilitation on overall cognition, attention, and executive function in individuals with chronic stroke compared with control groups. However, VR-based training combined with rehabilitation showed non-significant improvement in global cognition in patients with chronic stroke. The positive result of VR-based training on attention/execution and overall cognition of patients with stroke in the current meta-analysis was in consistence with data from a previous systematic review of mild cognitive impairment or dementia (Zhu et al., 2021). However, the negative result of VR-based training on global cognition of patients with stroke in the current meta-analysis was not in consistent with data from VR-based interventions on global cognitive function measured with MMSE or MoCA test in individuals with stroke (Aminov et al., 2018) and neurocognitive disorders (Moreno et al., 2019). The meta-analyses by Aminov et al. (2018) included 4 RCTs investigating the effect of VR-based rehabilitation in patients with stroke and reported small to medium effect VR intervention for cognitive outcomes. While the participants in 3 of the 4 included RCTs were at the subacute phase, 1 RCT did not report the time since onset of stroke before intervention. In terms of the severity for global cognition at baseline, 2 of the 4 included RCTs reported an average of mild cognitive impairment, and another 2 RCTs included an average of moderate cognitive impairment. The positive result of VR-based training on attention/execution of patients with stroke in our study was not in consistence with data from the previous meta-analysis (Wiley et al., 2020), while the negative result of VR-based training on global cognition of patients with stroke in our study was in consistent with data from previous meta-analyses (Wiley et al., 2020; Zhang et al., 2021). Wiley et al. (2020) performed a meta-analysis that included 5 RCTs to investigate the effectiveness of VR-based rehabilitation on global cognition and specific domains of cognition such as memory, attention, and language. However, the participants in 2 of the 5 included RCTs were at the subacute phase, 2 RCTs were at the chronic phase, and 1 RCT was at the subacute or chronic phase. In terms of the severity for global cognition at baseline, 3 of the 5 included RCTs reported mild cognitive impairment, 1 RCT included reported moderate cognitive impairment, and 1 RCT included reported moderate or mild cognitive impairment. Besides, one study included (Oh et al., 2019) did not exclude individuals with visual impairment. A meta-analysis by Zhang et al. (2021) included 7 RCTs using MMSE for measuring cognitive function, and the result showed no significant difference in cognitive outcomes after the VR interventions compared with control groups. However, for PEDro Scale scores, 5 of the 7 included RCTs in this meta-analysis were of low quality, and 3 of the 7 included RCTs were not present in the reference. In terms of the time since onset of stroke before the intervention, participants in 2 of the available 4 included RCTs were at subacute phase, 1 of the available 4 included RCTs was at chronic stage, and 1 of the available 4 included RCTs was at the subacute or chronic stage.

The results of the limited current review provided evidence that VR could not contribute to motor rehabilitation. In this meta-analysis, 3 of the included 5 RCTs measured motor function (upper extremity motor function), while two included RCTs (Oh et al., 2019; Maier et al., 2020) used VR device-based cognitive rehabilitation and one included RCT (Faria et al., 2018) used VR device-based cognitive-motor task. Due to the small number of participants, different VR devices aiming at improving cognition, and the different scales measuring motor function in the included studies, our analyses failed to find positive results. However, cognition and motor function potentially influence each other in many ways, indicating that motor recovery might positively affect cognition. Previous studies have shown the potential benefits of VR-based motor rehabilitation on cognitive and motor outcomes (Gamito et al., 2017; Manuli et al., 2020). Furthermore, VR-based cognitive-motor intervention systems are encouraging (Faria et al., 2018; Kannan et al., 2019; Manuli et al., 2020).

Improvement in ADLs was included in two of the studies. In one included study (Manuli et al., 2020), the FIM and SF-12 increased significantly in accordance with the improvement in cognitive and behavioral outcomes, indicating better QoL after the VR-based treatment. However, Faria et al. (2018) reported no significant difference in improvement for BI along with cognitive function. This potentially indicates that cognition and participant may influence each other. Poor performance in motor activities and poststroke cognitive impairment can lead to depression, anxiety, and impairment of social functioning (Kim et al., 2019). Two studies in this review reported that either motor-based VR training or cognitive-based VR intervention indicated a positive effect on cognitive abilities and improvement in motor functions with a reduction in a depressive mood. Furthermore, mood symptoms of patients should be examined in future studies for VR intervention.

The controversial findings for VR-based training for cognitive and motor function may be related to the following aspects. First, insufficient VR programs designed for cognitive function training might be used. Second, this might be related to the wide range of training duration. Third, the different assessment tools for cognition after stroke were used. Finally, the time since onset of stroke before intervention largely varied from the acute phase to several years after stroke.

While the VR-based intervention was argued not to be exclusively dependent on a higher dose, frequency, or daily intensity (Muratori et al., 2013; Gamito et al., 2014; Laver et al., 2015; Winstein et al., 2016b), the subgroup analysis of our meta-analyses found more positive effects of a greater dose of VR therapy (more than 20-h intervention), higher frequency (more than four times per week), and higher daily intensity of VR therapy (more than 60 min per day) in the recovery of cognition, motor function, mood, and ADL. These findings differed from the recent meta-analysis of VR studies. Positive results were seen (Palma et al., 2017) for VR interventions using immersive, semi-immersive, and non-immersive environments for patients with subacute and chronic stroke with a mean dose of 17.6 h for upper extremity and 13.2 h for motor function. Aminov et al. (2018) reported a mean daily intensity of 42 min and weekly intensity of 153.9 min, a mean frequency of three sessions a week and a median duration of 18 sessions, and a total of approximately 12 h VR interventions and found that VR showed no advantage for a greater duration, higher doses, or massed training schedules in individuals with stroke compared with the control group. However, in the review by Aminov et al. (2018), the duration of stroke before intervention was not mentioned in one of the 4 included RCTs measuring cognitive outcomes, three of the included RCTs reported inclusion criteria for patients with subacute stroke, only 2 included studies targeted cognitive function alone, and the other 2 studies also aimed to improve motor function. In this review, semi-immersive environments reported in 5 included RCTs were used for patients with chronic stroke. Large variability was found for the VR intervention parameters including RCTs, with studies providing up to 1.5 h intervention daily intensity, up to five times per week frequency, and up to 40 sessions duration.

The multisensory stimulation of head-mounted display (HMD) device-induced immersive VR training is thought to have a better effect on behavioral outcomes in patients with stroke. However, a recent study conducted by Gamito et al. (2014) revealed no significant difference between HMD-induced immersive VR and desktop screen-induced semi-immersive VR for increasing working memory and sustained attention in patients with stroke. The widespread use of HMD displays may have some limitations, and the HMD devices are more expensive than screens and often not portable, difficult to justify for lay users, and may cause visual discomfort. Thus, the non-expensive displays of semi-immersive environments of VR-based cognitive rehabilitation might provide a cost-effective schedule for patients with chronic stroke with cognitive and motor dysfunction.

### Strengths and Limitations

To the best of our knowledge, this meta-analysis was the first to explore the effects of VR-based therapy for cognition, motor function, depressive mood, and ADL in the chronic phase of patients with stroke. Specific cognitive domains and subgroup analysis including intervention frequency, intervention daily intensity, dose of intervention, and intervention sessions were also performed based on study characteristics. However, this review had several limitations. First, due to the less number of participants, the impact of stroke type and stroke locality and severities of stroke on the effect of VR-based rehabilitation could not be controlled. Second, all included studies used semi-immersive VR therapy systems, and no study used immersive VR therapy systems. Third, the limited number of RCTs may affect the effect of VR-based therapy and might be subject to potential publication bias. Although VR techniques were widely used in neurological diseases in the recent 20 years, only 6 RCTs were included in the current systematic review. Finally, future multicenter clinical studies are needed to investigate the long-term effects of VR-based rehabilitation on changes in neuroimaging biomarkers and neuroelectrophysiological mechanism.

### Conclusion and Implications for Practice

Our pooled data from the literature suggests that VR-based therapy combined with traditional rehabilitation showed better outcomes compared with traditional rehabilitation on overall cognition, attention, executive function, and depressive mood in individuals with chronic stroke. However, VR-based training combined with traditional rehabilitation showed a non-significant effect over traditional rehabilitation therapy on global cognition, motor function, and ADL for individuals with chronic stroke. Subgroup analysis for VR-based training suggested a greater intervention dose (more than 20 h of intervention), higher intervention frequency (more than four times a week), and daily intensity (more than 60 min of daily intervention) may be more advantageous for patients with chronic stroke to enhance their overall function, activity, and participant. Larger multicenter randomized trials determining the efficacy and effectiveness of VR-based therapy on cognition after chronic stroke are needed. Well-designed RCTs will advance our understanding on the dosage, frequency, and intensity of VR-based therapy for cognition in chronic stroke.

## Data Availability Statement

The original contributions presented in the study are included in the article/Supplementary Material, further inquiries can be directed to the corresponding authors.

## Author Contributions

YG, LM, CL, and HF conceptualized and performed the analysis. TW and JG supervised the study. YG, LM, and LLY drafted the manuscript. SZ, CL, and XY analyzed the data. YG and TW participated in the whole process and made final decisions. All authors contributed to the writing of this manuscript and read and approved the final manuscript.

## Conflict of Interest

The authors declare that the research was conducted in the absence of any commercial or financial relationships that could be construed as a potential conflict of interest.

## Publisher’s Note

All claims expressed in this article are solely those of the authors and do not necessarily represent those of their affiliated organizations, or those of the publisher, the editors and the reviewers. Any product that may be evaluated in this article, or claim that may be made by its manufacturer, is not guaranteed or endorsed by the publisher.

## Supplementary Material

Supplementary Appendix 1 Search strategies for web of science database.
