# Corrigendum: Effects of virtual reality-based intervention on cognition, motor function, mood, and activities of daily living in patients with chronic stroke: A systematic review and meta-analysis of randomized controlled trials

**DOI:** 10.3389/fnagi.2022.1128402

**Published:** 2023-01-24

**Authors:** Yong Gao, Lu Ma, Changsheng Lin, Shizhe Zhu, Lingling Yao, Hong Fan, Jianqiu Gong, Xiaobo Yan, Tong Wang

**Affiliations:** ^1^Department of Rehabilitation, Shaoxing People's Hospital (Shaoxing Hospital, Zhejiang University School of Medicine), Shaoxing, China; ^2^Library, Zhejiang Industry Polytechnic College, Shaoxing, China; ^3^School of Rehabilitation Medicine, Nanjing Medical University, Nanjing, China; ^4^Department of Rehabilitation, The First Affiliated Hospital of Nanjing Medical University, Nanjing, China

**Keywords:** cognition, motor, virtual reality, chronic stroke, meta-analysis

In the published article, there was an error in section **Materials and Methods**, Statistical analysis, Paragraph.

Instead of “*The effect size (ES) was categorized as follows: small (≤0.2), medium (>0.2 and ≤0.5), and large (>0.5),” it should be “The effect size (ES) was categorized as follows: small (<0.3), medium (≥0.3 and<0.6), and large (≥0.6)*.”

In the published article, there was also an error in section **Introduction**.

Instead of “*Basic neuroscience behind VR-based treatment was the finding of mirror neurons (MNs) in the primary motor cortex (M1), dorsal premotor cortex, supplementary motor area (SMA), and M1 from animal studies*,” it should be “*Basic neuroscience behind VR-based treatment was the finding of mirror neurons (MNs) in the primary motor cortex (M1), dorsal premotor cortex, and supplementary motor area (SMA) from animal studies*.”

The authors apologize for this error and state that this does not change the scientific conclusions of the article in any way. The original article has been updated.

