# Supplementary material for: Effects of Virtual Reality-Based Intervention on Cognition, Motor Function, Mood, and Activities of Daily Living in Patients With Chronic Stroke: A Systematic Review and Meta-Analysis of Randomized Controlled Trials
Source: Front Aging Neurosci. 2021 Dec 13;13:766525. doi: 10.3389/fnagi.2021.766525 (PMC8710683; doi:10.3389/fnagi.2021.766525)
Supplement: Supplementary Appendix 1 — Search strategies for web of science database. [file Table_1.DOCX]

**Web of Science search strategy**#1.TS=(stroke or poststroke or post-stroke or cerebrovasc* or brain vasc* or cerebral vasc* or cva* or apoplex* or SAH)
#2.TS=((brain* or cerebr* or cerebell* or intracran* or intracerebral) NEAR/5 (isch$emi* or infarct* or thrombo* or emboli* or occlus*))
#3.TS=((brain* or cerebr* or cerebell* or intracerebral or intracranial or subarachnoid) NEAR/5 (haemorrhage* or haematoma* or hematoma* or bleed*))
#4. TS=(hemipleg* or hemipar* or paresis or paretic or brain injur*）
#5. #4 OR #3 OR #2 OR #1

6. TS=(virtual reality or computer interface or computer or computer program or computer simulation or computer assisted therapy or computer graphics or touch)

7. TS= (virtual reality$ or virtual-reality$ or VR)

8. TS= (virtual NEAR/3 (environment$ or object$ or world$ or treatment$ or system$ or program$ or rehabilitation$ or therap$ or driving or drive$ or car or tunnel or vehicle))

9. TS= (computer NEAR/3 (simulat$ or graphic$ or game$ or interact$))

10. TS= (computer NEAR/1 assist* NEAR/1 (therap* or treat*))

11. TS= (computer NEAR/1 generat* NEAR/1 (environment* or object*))

12. TS= (video game$ or video gaming or gaming console$ or interactive game or interactive gaming or Nintendo Wii or gaming program$)

13. TS= (user NEAR/1 computer NEAR/1 interface)

14. #6 OR #7 OR #8 OR #9 OR #10 or #11 OR #12 OR #13

#15.TS=(random* or RCT or RCTs)
#16.TS=(controlled NEAR/5 (trial* or stud*))
#17.TS=(clinical* NEAR/5 trial*)
#18.TS=((control or treatment or experiment* or intervention) NEAR/5 (group* or subject* or patient*))
#19.TS=(quasi-random* or quasi random* or pseudo-random* or pseudo random*)
#20.TS=((control or experiment* or conservative) NEAR/5 (treatment or therapy or procedure or manage*))
#21.TS=((singl* or doubl* or tripl* or trebl*) NEAR/5 (blind* or mask*))
#22.TS=(cross-over or cross over or crossover)
#23.TS=(placebo* or sham)
#24.TI=trial
#25.TS=(assign* or allocat*)
#26.TS=controls
#27. #26 OR #25 OR #24 OR #23 OR #22 OR #21 OR #20 OR #19 OR #18 OR #17 OR #16 OR #15

#28.#26 AND #14 AND #5
